# Supplementary material for: Transcriptome Analysis Reveals Vimentin-Induced Disruption of Cell–Cell Associations Augments Breast Cancer Cell Migration
Source: Cells. 2022 Dec 13;11(24):4035. doi: 10.3390/cells11244035 (PMC9776984; doi:10.3390/cells11244035)
Supplement: Supplementary file 1 [file cells-11-04035-s001.zip › DEGs and enrichment analysis.zip!/DEGs and enrichment analysis/WT_VIMvsVector KEGG/ALL/WT_VIMvsVector_ALL.pathway_map.zip!/WT_VIMvsVector_ALL.pathway_map/src/hsa05224.html]

hsa05224


- hsa:5241

- Up regulated genes

ENSG00000082175(4.22083564138011)

- hsa:5241

- Up regulated genes

ENSG00000082175(4.22083564138011)

- hsa:1870

- Up regulated genes

novel.507(3.55620908288126)
- hsa:1869

- Up regulated genes

ENSG00000101412(1.977544660594)

- hsa:23493

- Up regulated genes

ENSG00000135547(3.89723839640576)

- hsa:2324

- Up regulated genes

ENSG00000037280(2.35155506151555)

Close
